# The 2018 Revision of Italian Dietary Guidelines: Development Process, Novelties, Main Recommendations, and Policy Implications

**DOI:** 10.3389/fnut.2022.861526

**Published:** 2022-03-25

**Authors:** Laura Rossi, Sibilla Berni Canani, Laura Censi, Laura Gennaro, Catherine Leclercq, Umberto Scognamiglio, Stefania Sette, Andrea Ghiselli

**Affiliations:** Council for Agricultural Research and Economics - Research Center for Food and Nutrition (CREA – Food and Nutrition), Rome, Italy

**Keywords:** dietary guidelines, nutritional recommendations, food policy, consumer behavior, household food safety, sustainability, Italy

## Abstract

The fourth edition of the Italian Dietary Guidelines (IDGs) for Healthy Eating was published in 2019. The objective of this paper is to describe the developmental process of IDGs, the main recommendations, the differences with previous revisions, and the concordance and differences with international guidance on a healthy diet. A National Commission oversaw IDG development. A Scientific Dossier (SD), including analysis on nutrition, health, and risk factors status in Italy, was the reference for IDGs preparation. The IDGs are based on the principles of the Mediterranean Diet and are mainly aimed to prevent obesity and nutrition-related non-communicable diseases. The IDGs included 13 directives that were divided into four conceptual blocks: i) how to balance weight; ii) foods to be promoted; iii) foods to be limited; and iv) how to ensure a varied and sustainable diet. Each directive has a box summarizing the key recommendation, myths lists, and false beliefs to be dispelled. The topics of sustainability and the correct approach to food supplementation and weight-loss diet were introduced in the present edition of IDGs. This paper contributes to the debate on the complexity of derivation of Dietary Guidelines and their adaptation to the national context.

## Introduction

**Figure 1:**
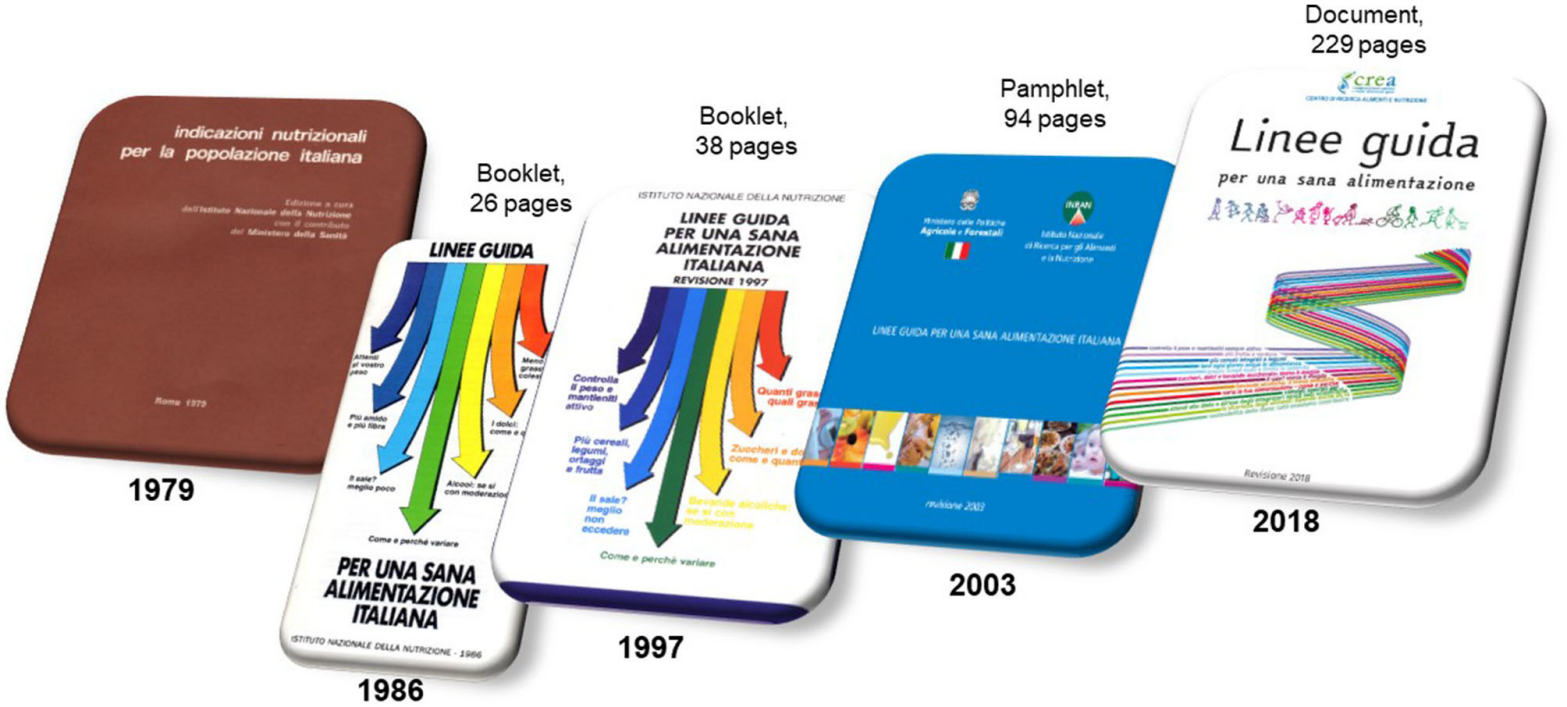
History of Italian Dietary Guidelines (IDGs).

The Research Center for Food and Nutrition of CREA (Council for Agricultural Research and Economics) in 2019 released the fourth edition of the Italian Dietary Guidelines for Healthy Eating– Revision 2018 (1). In Figure 1, the history of the Italian Dietary Guidelines (IDGs) is shown. The first exercise of the development of food-based nutritional recommendations in Italy was carried out in 1979 as a joint effort of the former National Institute of Nutrition and the Ministry of Health. This document defined the portion size to be guaranteed to a population that is still suffering from nutritional deficiencies (2). Proper dietary guidelines were first published in Italy in 1986 (3), followed by a second edition in 1997 (4), and the third one in 2003 (5). The IDGs have undergone these important revisions in terms of content, length, presentation style, and language, while maintaining relevancy and representing current scientific evidence regarding nutrition and health.

In Italy, the development, periodic revision, and implementation of the IDGs are an institutional task (defined by Law n.258/63; Law n.70/75; Legislative Decree 454/99) of CREA Research Center for Food and Nutrition (formerly National Institute of Nutrition). It is a consensus document accepted and endorsed by the scientific community and civil society stakeholders. As stated by DeSalvo et al. (6), the Dietary Guidelines are an important part of a complex and multifaceted solution to promote health and prevent diet-related chronic diseases, including cardiovascular disease, type 2 diabetes, some cancers, and obesity. Consequently, the IDGs are a public health document based on the health and nutrition epidemiological situation in Italy that proposes prevention strategies for the main critical diet-related public health problems. The IDGs were conceived to improve the nutritional quality of the diet, by modulating the quantities of food to be consumed and promoting lifestyle changes. As new topics of the last revision of IDGs, the improvement of the sustainability and environmental impact of the diet, and a specific chapter on food supplementation and correct approach to dieting was introduced.

The objective of this paper is to describe the developmental process of IDGs Revision 2018, the main recommendations, the novelties, the differences with previous revisions, and the concordance and differences with international guidance on a healthy diet as articulated by International Organizations, such as WHO and Food and Agriculture Organization (FAO) (7). This paper contributes to the research discussion related to the IDGs' evolving pathway and explains how the international recommendations were adapted to the Italian context. The research questions underlying this work are: i) to what extent is it possible to translate evidence from nutrient-based, food-based, and dietary patterns research in dietary guidelines? ii) to what extent is it possible to propose behavioral changes towards the desired recommendations, considering that the acceptance of new inputs in the diet require time? and iii) is it possible to evaluate the impact of dietary guidelines and what methodology could be proposed?

## The IDGs' Development Process and Policy Actions Implications

**Figure 2:**
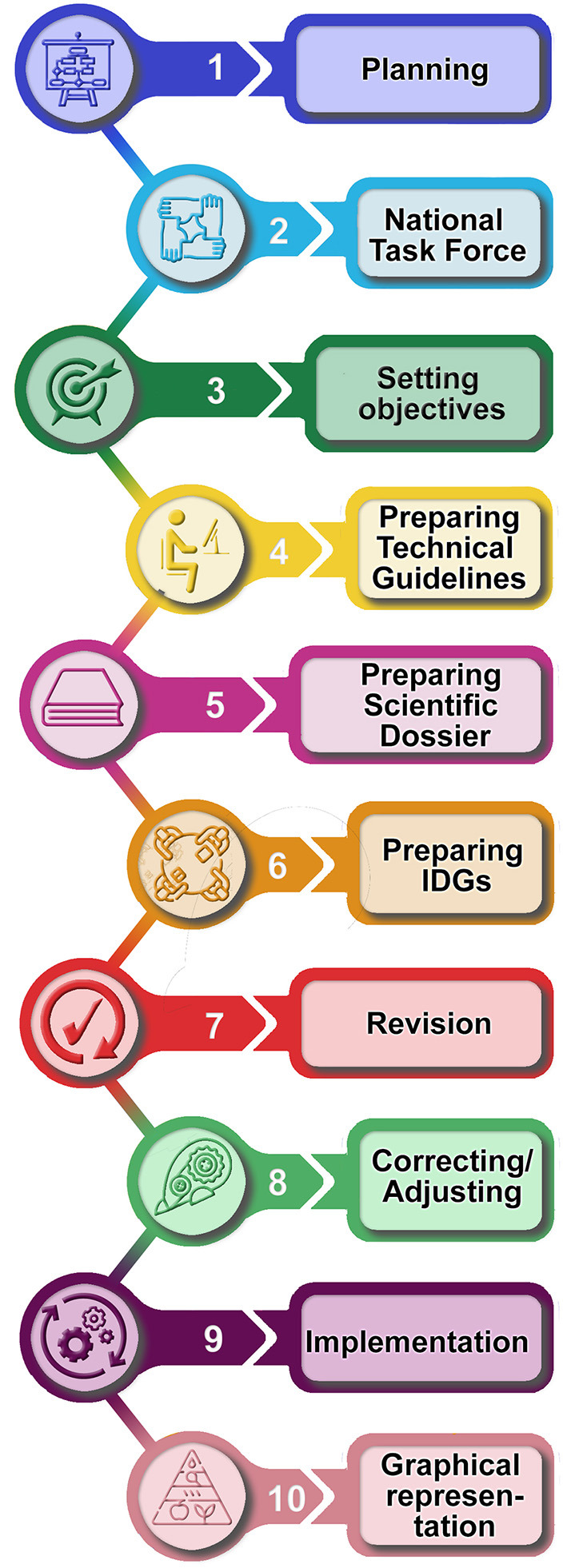
Steps of preparing the IDGs.

The steps of preparation of IDGs (Figure 2) started in 2013 with the appointment of the Commission of Revision of IDGs, a National Task Force, having the assignment to elaborate the Scientific Dossier (SD) (8). The IDGs' Commission of Revision composition is reported in the Supplementary Material. The nature of the commission, which included competencies in food and nutrition sciences from Academia and National Research Institutions, representatives of Scientific Societies (medical, nutrition, physical activity, obesity, and nutritional disorders), psychologists, consumers associations, representatives from Ministries of Health, Ministry of Environment, and Ministry of Education, is a guarantee of the consensual nature of IDGs. The work was coordinated by the Editorial Coordination Board that was responsible for SD evidence adaptation/translation in the IDGs for amending the document, and for preparing and editing the IDGs final version. An official endorsement from FAO and WHO was provided and was included as a preface of the final IDGs document. In 2017, the first version of the IDGs was released by the Editorial Coordination Board and revised by the IDGs Commission of Revision. The amended final versions of the documents [SD (8), IDGs (1)] were officially launched in 2019.

The SD (8) analyzed and evaluated the health and nutrition situation in Italy, the prioritization and determining strategies for alleviating public health problems, and the national goals to be reached through the IDGs. In addition to that, a description of Italian food consumption patterns and eating habits was carried out. In the SD, the key points to be included in the IDGs were identified as a general framework for the preparation of the policy document. The methodology used for SD was functional to the objectives of IDGs and was shaped accordingly. The study of the bibliography was focused on peer-reviewed papers, and complemented by the technical reports of scientific and governmental bodies that were included in the review based on the Authors' decision. The Commission of Revision decided that the so-called “grey literature,” i.e., papers under potential conflicts of interest or works published in reports or journals not subject to the external review procedure or private sector reports, was consulted and cited if the conclusions were also substantiated by the official literature. The literature review of the 13 chapters of the SD corresponding to the 13 directives of the IDGs was carried out using a set of keywords specifically reported for the different topics in a dedicated paragraph in each chapter of the SD (8). This work was based on consultation of the literature databases of the authors, combined with the search for published works using Medline, ScienceDirect, and Ingenta. The main inclusion criteria for bibliography selection were temporal with the consideration of papers published in the period 2000–2018. The older bibliographies were reported only in case of very consensual and generally accepted results. The focus of the literature review was on human studies rather than animal studies, metanalysis, and systematic reviews, to provide a robust statement as much as possible. The articles on animal and cellular models and case studies have been critically reviewed for the scope of the work and cited only if relevant. Approximately 5,000 references are cited in the whole SD (8). The construction of IDGs was based on the overall SD literature analysis, allowing a holistic approach to food and nutrition that included epidemiological and biomedical aspects together with sociocultural habits, behavioral attitude, and sustainability aspects.

### The Health and the Nutritional Situation in Italy

Italy, according to official OECD statistics (9), is ranked 25th (out of the 37 OECD member states in 2017) for the prevalence of overweight and obesity, with a worryingly high prevalence in childhood (10, 11). The poor adherence of the Italian population to the Mediterranean Diet, as well as the high level of sedentary lifestyle (12, 13), could both be among the main factors to explain the rise in the prevalence of obesity in Italy (14). According to the data reported by DaSilva et al. (15), further confirmed by Vilarnau et al. (16), many countries in the Mediterranean basin were drifting away from the Mediterranean dietary pattern. A recent review (17) has shown a high prevalence of children and adolescents living in southern European countries (Italy, Spain, and Greece) with poor adherence to the Mediterranean diet. The dietary shift of Mediterranean basin countries could have influenced nutritional outcomes, considering the higher prevalence rates of childhood overweight and obesity in the Mediterranean countries (i.e., Cyprus, Greece, Malta, Italy, and Spain), compared to other countries (18).

According to a study carried out by the Global Burden of Disease (GBD) Italy Collaborators (19), in 2017, the Italian life expectancy was among the highest globally, with life expectancy at birth reaching 85.3 years for females and 80.8 years for males. Among nutritionally related disorders, cardiovascular diseases decreased by 53.7% and neoplasms decreased by 28.2% in terms of age-standardized death rates in 1990 and 2017. Behavioral nutritional risk factors, which are potentially modifiable, still have a strong effect, particularly on the mentioned diseases, and considering the aging of the population. For instance, in 2017 in Italy, 12,000 deaths were attributed to alcohol use, and 9,500 to high body mass index (BMI), while 47,000 deaths due to cardiovascular diseases could be attributed to high Low Density Lipoprotein (LDL) cholesterol, 28,700 to low whole grains consumption, and 15,900 to low physical activity. On the other hand, a high intake of sodium, low intake of whole grains, and low intake of fruits were the leading dietary risk factors for deaths and disability-adjusted life-years (DALYs) globally and in many countries (20).

We mentioned the importance of IDGs as public health documents, but IDGs are also used as food policy documents aimed to improve the food behaviors of populations. According to FAO (21), dietary guidelines are intended to establish a basis for public food and nutrition, health and agricultural policies, and nutrition education programs to foster healthy eating habits and health-protecting lifestyles. They provide advice on foods, food groups, and dietary patterns to assure the coverage of nutrient requirements for the public to promote overall health and to prevent chronic diseases. The healthy food consumption patterns accomplishing these characteristics can be promoted through the development of Food-based Dietary Guidelines (21), which helps to maintain the high consumption of local and culture-specific foods (22).

The food consumption data at the national level (23, 24) provided information on dietary risk factors that required intervention for modification and correction. The observation of current Italian food consumption patterns showed that an improvement of the healthiness of the diet could be obtained through an increase in consumption of vegetable source foods, such as pulses, fruits, and vegetables, and through a decrease in consumption of red and processed meat. Italian diet nutrient intake excesses are related to fats (37% of total energy intake), saturated fatty acids (12% of total energy intake), free sugars (15% of total energy intake), and salt (10 g/die).

### The 13 Directives of IDGs

**Figure 3:**
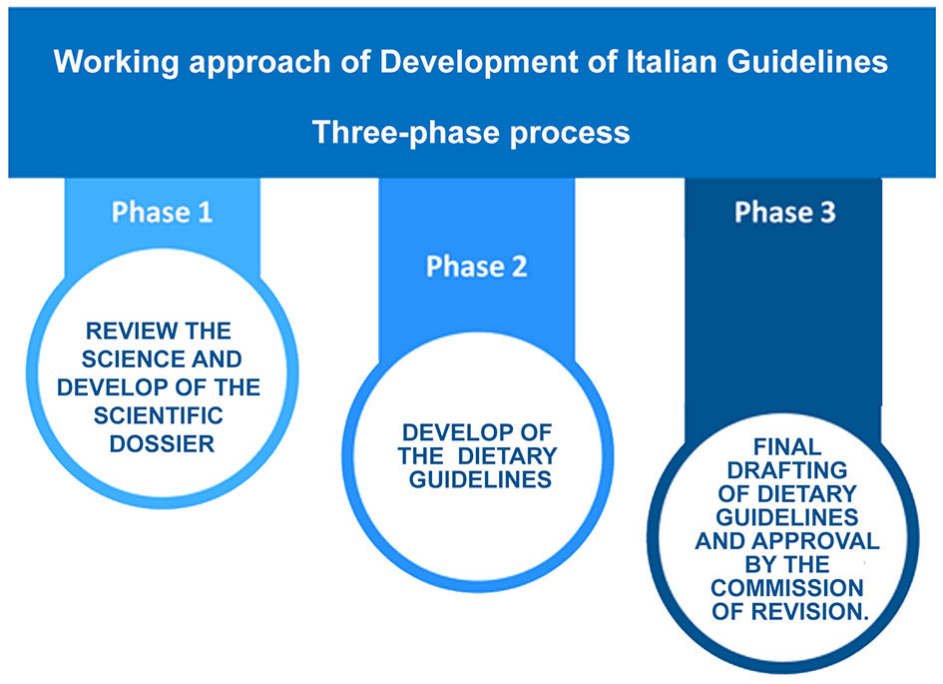
Working approach of development of the IDGs.

The working approach of IDGs was based on a three-phase process (Figure 3). The SD was used as the benchmark for the development of each chapter of IDGs, providing the emerging issues, and the identification of the main topics to be included in the 13 directives of IDGs. The second phase was characterized by broad discussions of the SD content by the Editorial Coordination Board to define the outlines of each guideline and how to convey messages and recommendations. The third phase was the final drafting of the IDGs and their approval by the Commission of Revision.

The IDGs are based on the principles of the Mediterranean Diet (MD), a model that has gained fame and honor, being the dietary pattern that combines prevention of non-communicable chronic diseases, longevity, and health, with consumers' acceptability and sustainability (25). The MD-related food products and eating habits can vary from country to country. The IDGs were developed to declinate the principles of the MD adapting them to the most recent international recommendations. The challenge of this adaptation is the identification of a local healthy food consumption pattern as part of the healthy lifestyle that the IDGs need to recommend.

The IDGs recommendations cover all age groups, from infants to the elderly, including the special needs of physiological conditions, such as pregnancy and lactation. There is also a focus on the requirements of people who practice sports, as well as recommendations for people at increased risk of obesity and most common non-communicable chronic diseases, such as cardiovascular and cerebrovascular diseases, diabetes, and cancers.

Table 1 reports the titles, the summary of the content, and the main recommendations of the 13 directives that were divided into four conceptual blocks. The first block is related to balancing weight, food intake, and physical activity (Directive 1). The second block is dedicated to food categories that need to be promoted to increase consumption, such as fruits and vegetables, legumes, whole grain cereals, water (Directives 2–4). The third block concerns critical food components in the current diet that need to be reduced, such as fat, salt, sugar, and alcohol (Directives 5–8). The last block is dedicated to “How to” ensure a varied, safe, healthy, and sustainable diet (Directives 9–13).

**Table 1:** The 4 conceptual blocks, the 13 directives, and the main recommendations of each directive.

**Conceptual block**	**Directive**	**Main recommendations**
Balance	D1. Keep your weight under control and always be active	• In the case of overweight reduce food intake and increase physical activity. • Avoid very restrictive diets that exclude entire food groups. • Be careful to extreme food behavior that could be symptoms of eating disorders.
Increase consumption	D2. Eat more fruits and vegetables	• Increase fruit and vegetable consumption limiting the adding of added fats and salt. • Choose seasonal fruit and vegetables in varying colors. • Fruit juice cannot replace a portion of fresh fruit.
	D3. Eat whole grains and legumes	• Increase consumption of fiber by choosing whole grain products. • Increase legume intake as an alternative to animal source food. • Remember the importance of whole grains as protective factors for non-communicable diseases.
	D4. Drink abundant water every day	• Water must be the preferred fluid for rehydration. • Drinks at least 8 glasses of water a day, more is better. • Increase water intake during physical activity.
Reduce consumption	D5. Fats: select which ones and limit the quantity	• Reduce intake of saturated fat choosing foods containing unsaturated fatty acid for cardiovascular prevention. • Remember that all fats have the same caloric content. • Remember that in Italy trans-fatty acids are no more used in industrial products.
	D6. Sugar, sweets, and sugar-sweetened beverages: less is better	• Reduce intake of sugar in favor of starchy foods. • A high intake of sweetened beverages is a risk factor for non-communicable diseases, including diabetes and obesity. • Remember that brown sugar, honey, and fructose are not healthy alternatives to sugar.
	D7. Salt: less is better (but iodized)	• Reduce intake of salt and choose iodine-fortified products. • Remember that several industrial products are hidden sources of salt (e.g., breakfast cereals). • Remember that salt intake is an important risk factor for non-communicable diseases in particular heart disorders.
	D8. Alcoholic beverages: the least possible	• Avoidance of alcohol from any source, including wine and beer, is the best for health. • If you decide to drink alcohol it is for your pleasure not for health; limit the quantities: no more than 1 alcoholic unit (e.g., a glass of wine) per day for females and elderly and 2 alcoholic units per day for males. • No alcohol of any type to children, adolescents, pregnant and lactating women.
How to do	D9. Enjoy a variety of food choices	• Remember that choosing a variety of foods is a way to guarantee nutritional adequacy. • Variety does not mean more foods. Portions and frequencies must be adequate to energy consumption at different ages and physiological statuses. • Mediterranean Diet is the dietary pattern that inspires the Italian Dietary Guidelines.
	D10. Follow special recommendations for target groups	• Remember that children have special needs during infancy: select foods of high quality in adequate quantity. • Pregnancy and breastfeeding are physiological periods that require attention: best to think about that before, to arrive at these moments in good health and put in place all preventive actions needed (e.g., folic acid supplementation). • The elderly need to eat a little less because the energy metabolism slows down, but the quality of the food must be higher, without forgetting to maintain an active lifestyle.
	D11. Be careful of dieting and misuse of dietary supplements	• Dieting is a therapeutic act that requires trained professionals; consumers should avoid referring to non-qualified people. • Losing weight requires time and constancy, “everything and immediately” is not compatible with dieting. • Dietary supplements could be important in case of deficiency but never substitute a healthy diet.
	D12. Food safety depends also on you	• At home, be careful to adequately store food in the refrigerator. • At the supermarket, in the grocery cart and bags separate fruit and vegetables from meat, poultry, and fish to avoid cross-contamination. • Prepare the kitchen, clean the sink before and after washing and preparing fresh fruit and vegetables, use different cutting boards and preparation areas for meat/poultry/fish and fresh fruits and vegetables. Wash especially well between preparation of meat/poultry/fish and preparation of food that will be eaten without cooking.
	D13. Select a sustainable diet	• Avoid processed meat and reduce red meat consumption in favor of poultry or vegetal source of protein. Select fish from sustainable stocks, e.g., small fish from the Mediterranean Sea (anchovies, sardines, mackerel, etc.); do not demonize aquaculture. • Increase consumption of plant food avoiding the selection of products that require large use of external inputs for growing (e.g., high fertilizing, artificial light, and heating or overseas products). • Planning, preparing, and storing food can help consumers to waste less food, save money, and eat healthier food. Re-use old ingredients or leftovers in new dishes.

Each directive, as incipit provided “key recommendations” and practical and simple application behaviors that help consumers to put in place the advice. In addition to that, at the end of each directive, a list of “myths” and false beliefs was provided with the purpose to fight against fake news common in nutrition (26).

In terms of content, the overall philosophy of IDGs is the promotion of a plant-based diet that includes a large proportion of fruit and vegetable, whole grain cereals, and vegetable sources of proteins (legumes). Reduction of consumption of salt and sugar, and selection of animal source foods that combine health and environmental protection, such as milk, eggs, fish, and white meat, was also recommended. The importance of considering the whole dietary pattern, as protection for health and environment concerning single food or ingredient, was the key point of the present revision of IDGs. The bad/good dichotomy of food classification was avoided since it is considered too much reductive and simplistic.

### The IDGs Recommendations: What and How Has Changed From the Previous Version

Comparing the present IDGs with the version published in 2003, it is possible to identify, among others, a general change of the structure, starting with the directive's numbers that increased from 10 to 13. The 3 additional directives are: i) Directive 2 on fruit and vegetables; ii) Directive 11 on diet and supplements; iii) Directive 13 on sustainability. In addition to that, the IDGs document was overall updated, considering the new scientific data and the emerging aspects of public health nutrition. Each directive changed accordingly, providing updated recommendations. The newest aspects of the present version in comparison with the 2003 edition are: i) the recommendation on red and processed meat; ii) the position on alcoholic beverages; iii) the definition of portions size for the different classes of ages (infants, children, adolescents, and adults). In addition to these major changes, several other aspects of novelty were introduced in this revision of IDGs. As non-exhaustive examples, we can report the division in conceptual blocks that were never used before: the introduction of the concept of “discretionary foods” or “non-necessary foods”. The recommendations for people practicing sport were added in the present revision of IDGs. Each directive included recommendations for the prevention of obesity, cardiovascular disease, diabetes, and cancers, and these subjects were newer approached in this form in the previous revisions of IDGs. Also, the provision of “key recommendations” at the start of each chapter was a change introduced in the present revision of IDGs, as well as several other modifications that would be difficult to list in detail.

### Directive 2 – Fruit and Vegetable Promotion

In the published IDGs in 2003, fruit and vegetable recommendations were included in a unique directive promoting cereals, legumes, and other plant-based foods. Considering the growing evidence on the specific role of fruit and vegetable consumption in health promotion (27), in the present version of IDGs, a directive was specifically dedicated to these foods. The purpose of Directive 2 on fruit and vegetables was to stimulate their consumption, with the recommendation of multiplying the occasion of consumption to increase the quantity, to reduce the caloric density of the meals, and to increase the intake of health-protective components. Identifying any products better than others was avoided (e.g., red fruits or green vegetables), with the message that all fruits and vegetables are protective of health. It was clearly stated that the single component of fruits and vegetables, in pills or as extracts, do not have the same actions of eating these foods as part of a healthy diet. The fruit juices topic was treated in Directive 2 reporting the non-equivalence with fresh fruits and adding a table explaining the differences among the various commercial drinking-fruit products. Another concept of Directive 2 was that the ready-to-use and minimally processed products could be an option to increase the consumption of fruits and vegetables by the consumers who considered the preparation of these foods time-consuming.

### Directive 11 – Losing Weight Diets and Dietary Supplements

The introduction of Directive 11 was an absolute novelty in the IDGs, and the Commission of Revision discussed extensively the opportunity to include this directive since dieting is a therapeutic approach for overweight and obese people, while the IDGs refer to the general healthy population. However, it was considered important to approach this aspect in a public health document that would potentially reach a very large audience. The purpose of this directive was to counteract the growing myths and fake news that the so-called “diet industry” contributes to spreading out. The Editorial Coordination Board considered it important to address the topic of dieting in a policy document as IDGs in consideration of the possible fragility of the consumers that need to lose weight and could be exposed to the persuasion of non-scientific methods. The most common losing weight diets (low fats, low carbohydrates, ketogenic, Paleolithic, vegan, vegetarian, etc.) were analyzed showing the strengths and weaknesses of the different dietetic patterns. The final messages were that short-cuts and miracle diets do not exist and that to lose weight, it is necessary to reduce food intake and to increase physical activity. The topic of dietary supplements was also addressed; either considering the slimming products or other dietary pharmaceutical and herbal products. The final messages were that a supplement could not replace a healthy and balanced diet and that supplementation with natural products or with vitamins and minerals is not necessarily always safe, considering that excesses of ingestion could be not exempt from risks.

### Directive 13 – Sustainability of Food Choices

In line with the international indications (28), the issue of the environmental impact of food consumption and dietary choices was addressed in Directive 13. Existing food systems are dysfunctional and do not ensure access to healthy diets, being focused on providing abundant and cheap calories *via* mass production of staple commodities. Therefore, unhealthy dietary patterns and obesity spread up together with increased diffusion of socio-economic inequalities. Food system-related policies, such as agriculture, trade, food safety, environment, development, research, education, fiscal and social policies, market regulation, food waste management, and more, have been developed independently over decades, with minimal attention to dietary impacts (29). With Directive 13, the IDGs intended to contribute to bridging the cultural distance between nutrition and environmental aspects. The objective of the sustainability of the Directive of IDGs was the promotion of healthy food choices for the environment and social protection, and to counteract the food access and distribution inequities. Food waste was largely addressed in Directive 13, in consideration of the fact that since 2016, Italy endorsed the international commitment on this aspect with regulatory efforts (30) for the establishment of the National Observatory on Food Surplus, Recovery, and Waste (OERSA), aiming to collect data and carry out educational programs and awareness campaigns (31). In Directive 13, recommendations were provided for planning, preparing, and storing food that can help consumers to waste less food, to eat healthy and safe food, and save money. Re-use of leftovers in new dishes was suggested, also providing practical examples. An important message of Directive 13 was that a healthy diet is not necessarily a costly diet. Examples of simple, cheap ingredients with high nutritional values, such as eggs, poultry, beans, milk, and seasonal fruit and vegetables, were provided to guide consumers with limited income.

Another important difference with the previous versions of the IDGs was the introduction of the concept of “discretionary foods” or “non-necessary foods” that is different from the “basic foods”. The “discretionary foods” are commonly used foods, but not essential to meet macro and micronutrient needs. For most of them, it was not possible to define consumption frequencies in line with health-promoting aspects. In most cases, the IDGs recommended an occasional consumption that is limited to particular events as a pleasurable, social, and tasty moment. Included in this group, among others, are sweetened beverages, chips, ice cream, creamy tarts, processed meat, and alcoholic beverages.

### Recommendation on Red and Processed Meat

The development of recommendations on red and processed meat was based on the World Cancer Research Fund (WCRF) (32) recommendations that claimed for a limitation of the intake of red meat (no more than three portions per week of red meat corresponding to 350–500 g cooked weight) and consumption of very little, if any, processed meat. The importance of such recommendation was reinforced by the publication of the Monograph of the International Agency for Research on Cancer (IARC) (33), where processed meat is classified as “carcinogenic to humans” (Group 1) since sufficient evidence is available for colorectal cancer risk. In the same monograph, the red meat was classified as “probably carcinogenic to humans” (Group 2A) (34). In consideration of the IARC data solidity, which is also confirmed in Italian consumers cohorts (35–37), the recommendation of IDGs was to limit the consumption of red meat to one portion (100 g) per week, with the suggestion of replacing it with poultry that should be the preferred typology of meat (up to three portions per week). The processed meat was considered among the “discretionary foods”, and the recommendation was an occasional consumption in the quantity that is as little as possible. In Italy, at the disseminative level, the pork is frequently not considered red meat, while it was necessary for the IDGs to define the term “red meat” that, following IARC (33), refers to beef, pork, horse, lamb, and goat from domesticated animals.

### The Position on Alcoholic Beverages

The position on alcoholic beverages drastically changed in the last 20 years. The work carried out by the GBD 2016 Alcohol Collaborators (38) estimates the alcohol use and alcohol-attributable deaths and DALYs for 195 locations from 1990 to 2016. It provided very strong recommendations reporting zero standard drinks per week, as the level of alcohol consumption minimized harms across health outcomes. In addition to that, the WCFR/IARC (32) positioned alcoholic beverages within Group 1 of carcinogens, which is the group that specifically contains substances (and food items) “known to cause cancer in humans”. Considering these solid positions, the IDGs “shifted” from the previous suggested/recommended low-to-moderate alcohol consumption, to discouraging any level. This shift is mainly driven by epidemiological observations on alcohol intake-cancer association. The present IDGs considered that the evidence regarding the protective effects of low alcohol intake on cardiovascular diseases in specific segments of the population have been progressively considered less relevant and are negatively counterbalanced by the carcinogenic risks, at any level of consumption, when applied to the whole population as reported by WHO (39), with the consequent strong recommendation of avoidance of alcohol consumption to prevent cancers (40). This novel position has been endorsed by the panel of experts who drafted the IDGs intending to achieve the maximum protection of the population from risk factors. This position is common in the Food-based Dietary Guidelines in Europe (41), in which the messages related to alcohol consumption are very restrictive in large parts of the countries. The recommendation is “do not drink alcohol” without any orientation to healthy quantities. Similarly, when tolerated quantities are provided, it is specified that this “should not be taken as an encouragement to regularly consume alcohol”.

The IDGs considered alcoholic beverages in the group of non-necessary foods, stressing the harms of their consumption, and, consequently, with the indication of non-recommended food. However, we considered the role of this product in the Italian cultural background. Stressing the message that “There is not a quantity of alcohol exempt from harm” and “you better not drink”, it was also said that “If you want to drink, do it in strictly controlled quantities, and do it occasionally”. This indication may appear not sufficiently strong, as it leaves to consumers the decision to drink alcohol or not. Indeed, this is a compromise justified by the context of recommendations that address the population, avoiding being too strong taking into account the generalized traditional consumer habits.

### The Definition of Portions' Size for the Different Classes of Ages (Infants, Children, Adolescents, and Adults)

The IDGs contain diet plans, including low-calorie patterns, which include foods from all food groups using the Italian standard portions as a reference. The concept of portions and the importance of their knowledge by consumers is of fundamental importance for a balanced diet and was also present in the 2003 revision. In the present IDGs edition, the topic was broadened by defining the entity of the portions of the different foods, as well as their frequency of consumption to have a complete and balanced diet with foods of common use that are easily available and adhered to Italian culture and tradition. Completely new in this revision is the introduction of practical recommendations of consumption profiles that is also for infants, children, and adolescents, to help families organize a varied and balanced daily diet for these age groups. The Editorial Coordination Board considered it important to address this topic that was not present in the 2003 revision and is largely requested by technical operators (e.g., school canteens), consumers, and communicators. The quantities of different foods were adapted to make them suitable for children and teenagers starting from the portions defined for adults. Weekly menus differentiated by age range of three years where provided for the age groups from infancy to adolescence, to facilitate parents' food choices either in terms of quantity or quality.

Considering the above-reported modifications of the present version of IDGs, it is evident that the effort of the commission to align the directives with international recommendations also regarding traditional foods. For some directives, an equilibrium had to be found between the current Italian traditional food habits and the international guidelines. This effort led to some discussion within the commission, in which some members claimed a certain autonomy of a Member State in the development of its guidance, especially in the case of international recommendations, which appeared to be too far from the consumers' habits. In the IDGs' development, this criticism emerged for processed meat recommendations that some members considered too strong for Italian dietary habits. Another aspect that created discussion by some members was related to wine that the commission considered a source of alcohol, which is independent of its composition in terms of polyphenols, as well as independently from its consideration as food characterizing the Mediterranean diet. Another point that was discussed by some components was related to the fact that no mention was carried out to local branded products, such as the food with Protected Designation of Origin (PDO) or Protected Geographic Indication (PGI), while the position of the commission was to avoid the promotion of specific products or food chains. Considering the size of the commission, it was physiological to have different positions in the light of the backgrounds of the commission members that include agricultural sciences, nutritional sciences, epidemiology, and public health. The role of the coordination board was to synthesize the positions and, in some cases, to take decisions.

## Actionable Recommendations

The IDGs are intended to be used by health professionals who deal with nutrition, the private sector, and, in general, by the world of communication, generalist, and scientific. In addition to that, the main messages could represent the basis for school nutrition education programs.

The IDGs are used as the starting point to create training and message dissemination. Since the IDGs publication, participation in congresses, thematic conferences, bilateral informative meetings with the private sector, and training courses for nutritionists were carried out to disseminate IDGs concept, development process, general philosophy, novelties, and main recommendations.

### Training

Training packages for university students were structured and introduced in the nutritional science academic courses (42–47). All these academic efforts cover the nutritional education of more than 600 students per year but only in the areas of Rome (Italy). The IDGs were transformed into online training courses for nutritionists, medical doctors, and other health professionals providing formative credits (48, 49).

The IDGs are also used in-school educational programs as discussion material and information tools in teachers' training, and as the base to create playful and experiential laboratories targeted to children (50–52).

### Dissemination to the Public

For the general population, the IDGs were translated into short videos (3–4 min for each directive) published on a dedicated channel of the CREA's YouTube section (53). In addition to that, either as videos or as printable downloading text, the IDGs are included as informative material in the institutional website *sapermangiare.mobi* (54).

**Figure 4:**
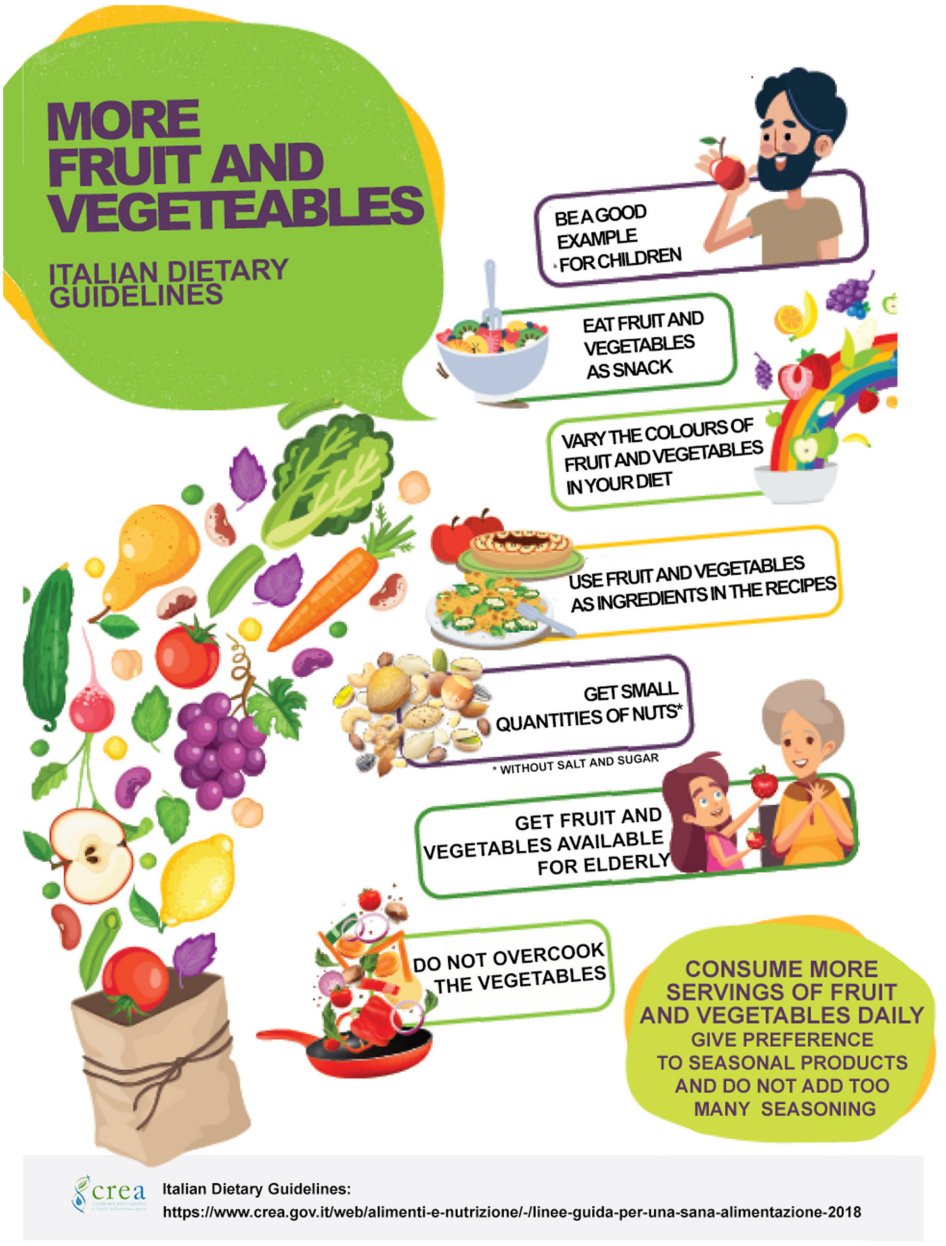
The decalogue for promotion of fruit and vegetable consumption.

As a further simplification, Directive 2 became a “Decalogue for promotion of the consumption of fruit and vegetable” (Figure 4), targeted to children and families, distributed in all fairs and events, where pupils and parents are present, and added to the textbook for teachers (55).

**Figure 5:**
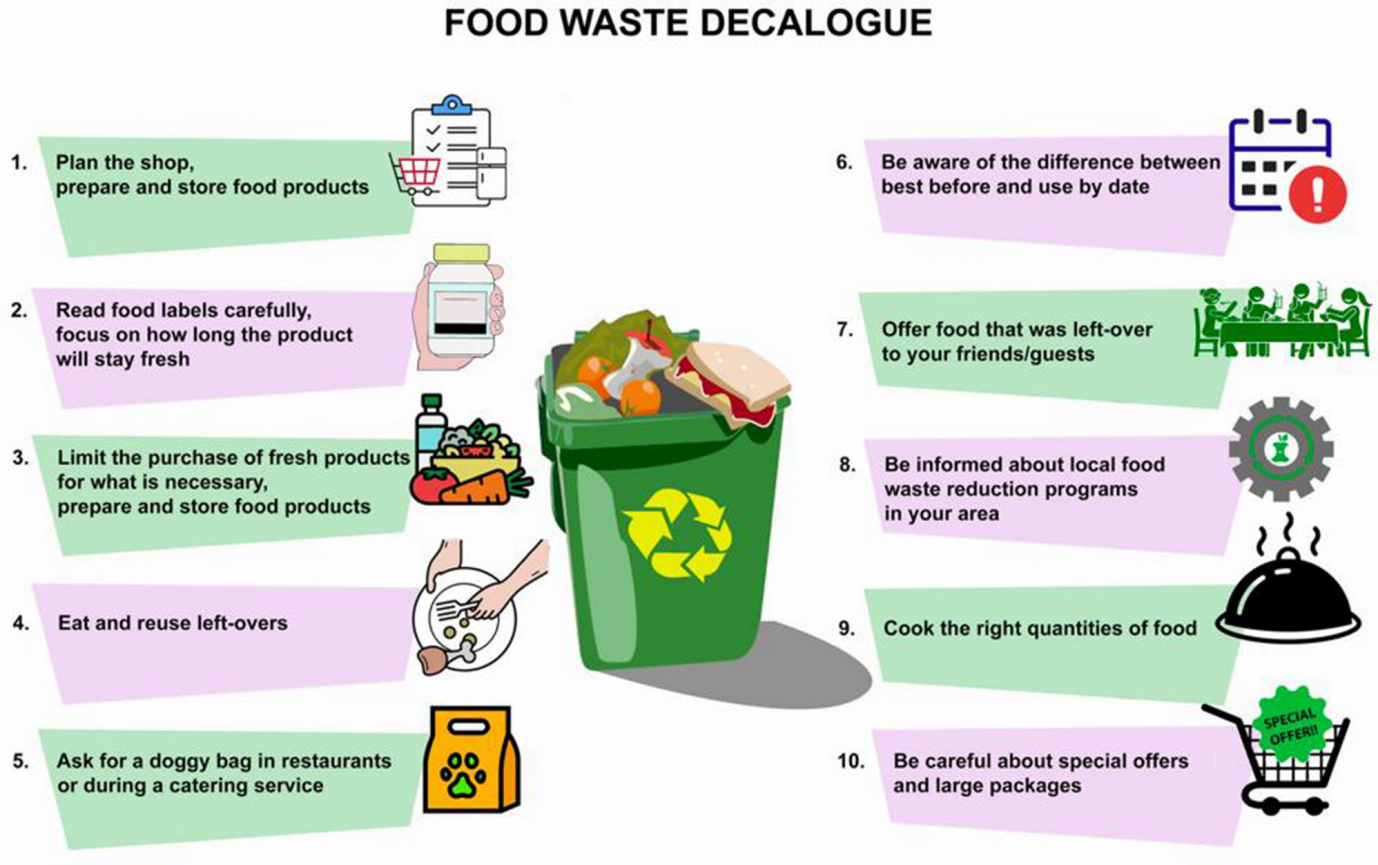
The decalogue against food waste.

With the same purpose from Directive 13, it was developed as “Decalogue against food waste”, which is a set of 10 recommendations (56) addressed to consumers that could be disseminated in schools, events, and information sessions. The 10 recommendations summarize the messages of Directive 13 simply and practically, pointing out the importance of: (1) reading labels by being aware of the difference between “best before” and “use by” date on the planning of food purchases and storing especially for fresh products, use of leftovers; (2) avoiding the stigma of asking for a doggy bag in restaurants and food catering; (3) offering foods that were left-over to guests, especially in the occasions that involve children to pass the message of not wasting food to youngest (Figure 5).

Further implementations of IDGs will be carried out according to the availability of resources. Starting from the published policy document, the extracts and synthetic information brochures will be structured for different targets, adults, adolescents, and children. The communication professionals will be used to the productions of leaflets to translate the scientific language into highly usable messages addressed to non-specialistic audiences that include consumers, as well as other interested stakeholders. These dissemination documents need to be graphically attractive, e.g., using infographics duly conceived by specialistic graphics service. The translation of the main recommendations of the IDGs in English may be considered for the dissemination of the document through international channels.

Professional short films (e.g., 100 s of guidelines) will be structured with infographics intended for the web to further disseminate the messages of the IDGs.

### Monitoring and Impact

Based on the available resources, the monitoring and impact evaluation will be carried out. The evaluation of dietary quality indices will be considered especially in terms of longitudinal adherence to the Mediterranean diet considering that Italian IDGs are based on Mediterranean Diet principles. The impact assessment of the nutritional education actions based on IDGs will be carried out through the development of the Nutrition Knowledge tool (57) that could be applied to different age groups through adaptation of the international questionnaires to the Italian context, comparison with eating habits, creation of indexes of adherence to recommendations, and evaluation of nutrition literacy in the general population and selected groups. This will allow the in-house data flow to monitor trends and to evaluate changes regarding the effects of IDGs implementation.

## Discussion

Dietary guidelines in general, and IDGs in particular, established a basis for public food and nutrition, health, and agricultural policies, and for nutrition education programs to promote healthy eating and lifestyles in the general population (58). In this paper, we described the process of development of IDGs to share with the scientific community the processes leading to its release, its adaptation to the national context, and the compromises that, in some cases, the commission accepted to pursue what was considered reachable to consumers' behavioral changes.

### The Evolution of IDG

IDGs key messages are aligned with recommendations of the WHO Healthy Diet Fact Sheet (58, 59) in terms of promotion of consumption of fruits and vegetables, legumes, whole grains, nuts; and limiting free sugars, salt, and fat, use of iodized salt. The recommendations of limiting red meat and avoiding processed meat and the consideration of alcoholic beverages as a harmful component of the diet were introduced in the present revision of IDGs to be in line with international recommendations. In fact, despite different geographical, socio-economic, and cultural contexts among countries, most of the pivotal nutritional recommendations are similar.

Since the first edition, IDGs changes reflected the best of nutritional science's available evidence. Without necessarily going through the text of the different revisions (in Italian), the idea of the changes is provided by the evolution of the titles of the directives of the IDGs across the years (Table 2). In the directive on bodyweight in 2003 and 2018, the physical activity recommendation was introduced in response to its increasing importance in the framework of bodyweight management (First line of Table 2). The number of directives was 7 in the 1986 and 1997 revisions and was increased to 10 Directives in 2003. The 3 directives added to the 2003 revision were related to the importance of water as a nutrient, and the correct hydration as an element of healthy eating. A directive on food safety in response to the momentum of the outbreak of several food safety emergencies (e.g., mad cow, dioxin chicken, etc.), and a directive aimed to provide specific recommendations for groups, such as pregnant and lactating women, infants and children, and the elderly, were introduced. In 2018, the overall number of directives further increased to 13 with the inclusion of directives on fruit and vegetables, which, in the previous revisions, were treated in a unique directive with whole grains and legumes, slimming diet and supplements, and sustainability, as presented in this paper. The language of the different IDG revisions evolved across the years. In the 1986 and 1997 editions, the prescriptive language was more common than in the 2003 revision and was further attenuated in IDGs published in 2018. For example, the directive on fats (Third line of Table 2) progressively become more qualitative, as the focus on cholesterol was eliminated in response to the literature evidence, and the combinations of qualitative and quantitative aspects were further added in recent years.

**Table 2:** Language, length, and recommendation changes of Italian Dietary Guidelines (IDGs) revisions since 1986.

**1986 edition** **7 directives**	**1997 revision 7 directives**	**2003 revision** **10 directives**	**2018 revision 13 directives**
Beware of your weight	Control your weight and stay active	Check your weight and always be active	Check your weight and always be active
More starch and more fiber	More cereals, legumes, vegetables, and fruits	More cereals, legumes, vegetables, and fruits	Eat more fruits and vegetables
			Eat whole grains and legumes
Less fat and cholesterol	How many fats, which fats	Fats: choose the quality and limit the quantity	Fats: select which ones and limit the quantity
Sweets: how and how many	Sugars and sweets: how and how many	Sugars, sweets, and sugar-sweetened beverages: within the right limits	Sugar, sweets, and sugar-sweetened beverages: sweets: less is better
Salt? Less is better	Salt? better not too much	Salt? Less is better	Salt? Less is better …(but iodized)
Alcohol: if yes with moderation	Alcoholic drinks: if yes with moderation	Alcoholic beverages: if yes, only in controlled quantities	Alcoholic beverages: the least possible
How and why to vary	How and why to vary	Often vary your choices	Enjoy a variety of food choices
		Drink abundant water every day	Drink abundant water every day
		Special recommendations for special people	Follow special recommendations for target groups
		Food safety depends also on you	Food safety depends also on you
			Be careful of dieting and misuse of dietary supplements
			Select a sustainable diet

A consumption pattern in line with IDGs reduces the risk of major chronic diseases by supplying adequate amounts of energy and nutrients, compared with the current food consumption intakes. The process of development of IDGs described in the present paper combined evidence-based elements with expert knowledge and common sense, as well as adaptation to local food consumption habits.

### IDGs Policy Implications

Aligning the dietary guidelines with the latest evidence, not just on healthy eating but also on the wider social and environmental implications of dietary choices is, therefore, an important starting point for enabling a policy coherence and building a food environment that contributes to good public and personal health, as well as to local and global environmental sustainability (60–62). The inclusion of sustainability in dietary guidelines is an open exercise that is still in progress in several countries. According to Springmann et al. (63), the inclusion of sustainability aspects into the national dietary guidelines, as well as in the WHO guidelines, could be beneficial not only from a health perspective but is also necessary for meeting global sustainability goals (64) and staying within the environmental limits of the food system. In IDGs, we demonstrated that it is possible to translate for consumers, the practical recommendations aimed to improve their behaviors in terms of environmental and social protection other than health. Probably, the strategy of prioritizing recommendations for health protection and combining them with environmental aspects avoided the confusion by being the most coherent public health nutrition document as IDGs. Considering that the IDGs' targets are families and individuals, even a few selected sustainability recommendations could have a large population impact. Limitations of this approach were largely discussed during the coordination meetings, along with the process of IDGs' development. The sustainability of diet is an aspect of the environmental impact of food production that is still not completely exploited, in which the risk of bias and “personal” interpretation is still high. At the time of IDGs preparation, the sources of information were limited, and the consensus documents were almost absent. As reported by Rosi et al. (65), the dietary recommendations, in terms of environmental impacts, should also consider the aspects related to the choice of locally grown and seasonal products, as well as agricultural and processing techniques, to approach globally the sustainability of the food system. However, it should be pointed out that the overall estimation of the different aspects of sustainability of the food system is still lacking.

The policy implications of IGDs are not different from other countries' nutritional guidance. A particularly relevant issue in this sense is how to properly communicate the information to the public in the current era of widespread and largely uncontrolled dissemination of information *via* an almost limitless variety of media outlets. Indeed, the nutritional issues, which are often intrinsically complex, are difficult to report comprehensively and, even when truly balanced, frequently fail in general communication and online communication (66). The long process of IDGs' development required cooperation among different Italian stakeholders, such as scientists, clinicians, and policymakers, who have an active part in the process. After the IDGs publication, to be maximally effective in terms of policy, the involvement of other society segments, such as the food private sectors, the communications areas, and the education sector, was carried out with different approaches such as evidence briefs, policy dialogues, and benchmarking. The Authors are conscious that these steps are just at the initial phases and that much more needs to be put in place.

### IDGs for Developmental Ages

According to Herforth et al. (67), the worldwide available Food-based Dietary Guidelines state that they apply to the general population: 46% qualify this statement with a “healthy” population and 13% refer to the general “adult” population. Over half (52%) specify an age above which the guidelines apply: almost all of these refer to age 2 years and older, although two countries, instead, specify 1 year, one country says 3 years, and three countries say 5 years. Forty percent of countries have separate messages or guidelines for specific subpopulations, which include infants under 2 years of age, school-aged children, adolescents, pregnant and lactating women, the elderly, and others. The IDGs' recommendations cover all age groups from infants to the elderly, including physiological conditions, such as pregnancy and lactation, to stay healthy during different periods of life. In this overall approach to all classes of ages, the Editorial Coordination Board decided to have a specific focus on infants, children, and adolescents providing practical recommendations with indicative serving sizes for consumption, expressed either as food items or food groups for developmental ages, to create menus coherent with the principle of a healthy and balanced diet. Another similar exercise was carried out by Kastorini et al. (68), that starting from Dietary Guidelines addressed to the general population, the developed food-based nutritional and physical activity recommendations for promoting healthy dietary habits in Greek infants, children, and adolescents proposed menus based on traditional Greek diet. Montagnese et al. (69) analyzed and compared the different European food-based dietary guidelines and reported that specific recommendations regarding children were provided in 21 countries (59%), while recommendations for adolescents were provided in 17 countries (50%). They pointed out that more emphasis should be given to some subgroups of the total population that currently represent a clear prevention target such as adolescents. Schwartz et al. (70) also evaluated the extended coverage of the international and national dietary guidelines of the themes of infants and children feeding habits. They concluded that guidelines, in general, cover most of the themes, but some of the national guidelines are incomplete. However, guidelines could be an occasion to give more practical tips to parents, especially to help them establish an appropriate feeding behavior for their children. According to UNICEF (71), only some of the guidance for specific groups are developed and disseminated as part of the national guidelines. The level of specificity in guidance for developmental ages varies greatly. For example, some countries provide very detailed guidance on how to initiate and maintain breastfeeding, how to choose and safely use infant formula, and how to introduce complementary foods. Relatedly, some newer DGs, including IDGs, also focus on the social role of meals in the family and the community, on the transmission of food skills to children and adolescents, and on the role of marketing and the need to limit the exposure of children to marketing, but also to educate children and adolescents on this issue. Some countries' DGs have also begun to address a wider range of behavioral issues around food and diets, including responsive feeding, parenting to help children develop healthy habits and healthy relationship to foods and eating, and addressing the developmental stage of adolescence. A specific chapter on adolescence was also included in the IDGs. The reasons for these differences are related to the fact that, especially in high-income countries, the government's guidance for specific groups (most commonly, infants, young children, and pregnant and lactating women) is developed through parallel processes and by specialized scientific societies, such as pediatrics, gynecologists, etc.

### The Limits of IDGs

The philosophy of IDGs is that the more plant-based a diet is, the more it is health-promoting and sustainable and that, among the different sources of proteins, red meat presence in the menu represents a critical issue both in terms of sustainability of the food choices and occurrence of non-communicable diseases. The IDGs consider that to promote human health and to reach an environmentally sustainable solution, the animal-based foodstuffs should be partially replaced with fruits, vegetables, legumes, and cereals, and among animal source foods, a selection of milk, eggs, fish, and white meat leads to better health promotion and environmental outcome in addition to being most in line with nutritional recommendations. However, such changes are hard to achieve at the population level considering that the diet to be promoted does not only depend on nutritional recommendations, but also needs to consider the social and practical aspects, cultural factors, and consolidated behaviors. The starting point of changes needs to be the current food consumption pattern of the population considering that large behavioral changes are difficult to be achieved. Indeed, advice towards drastic changes risks being ineffective. Instead, modulating the current consumption to achieve the nutritional and environmental goals step by step would result in a better outcome. The IDGs are important tools to inform policies and promote public health. To facilitate and improve adherence to IDGs and to have a real effect on food consumption, recommendations need to have clear links to Italian food policies.

It is widely recognized that even in high-income countries with a long history of developing, communicating, and otherwise implementing evidence-based DGs, the dietary patterns are far from ideal. It is important to acknowledge that while the development of DGs is necessary, which is to inform consumers and to program food and nutrition policy, their implementation is very far from sufficient (71). The main limitation of the IDGs is related to the existing gap between dietary recommendations and actual consumer behavior indicating the generally poor compliance of IDGs. An IDGs' implementation strategy is as equally important as the development of the evidence-based document. The need for effective communication to assist in translating the recommendations into practical and actionable advice is widely acknowledged and has been included as part of the global release of guidelines (72), even though in Italy, an articulated plan of developing strategies to assist behavior change is still lacking. The length of the process in developing and publishing the documents was another relevant limitation of IDGs. The question of whether a better compromise could be found remains open; for example, reducing the number of consultations. For example, discussions at the level of scientists that better know the literature could be reduced. On the other hand, probably, we should prioritize the consultations with stakeholders other than researchers to speed up the process.

### Conclusive Remarks

With this paper, we got the challenges of Bechthold et al. (73) that claimed efforts to have a common concept for the future derivation of European Food-based Dietary Guidelines. With the description of the Italian experience, we would contribute to the debate on the complexity of derivation of Dietary Guidelines, and their adaptation to the national context. We fully agree with the idea that a common European concept could serve as a starting point for the derivation of the national dietary guidelines that, however, need to be adapted to each country-specific condition. But to develop the common concept, we think that it is important to analyze the different experiences to get the lessons learned and to find cross-cutting recommendations to share, as well as valorize local peculiarities.

## Author Contributions

AG is the President and LR is the General Coordinator of the Commission of Revision of IDGs. SBC, LC, LG, CL, US, and SS are members of the IDGs' Editorial Coordination Board. The paper was conceptualized and writing and original draft preparation were carried out by LR. Writing, review, and editing were done by SBC, LC, LG, CL, US, SS, and AG. All authors have read and agreed to the published version of the manuscript.

## Conflict of Interest

The authors declare that the research was conducted in the absence of any commercial or financial relationships that could be construed as a potential conflict of interest.

## Publisher's Note

All claims expressed in this article are solely those of the authors and do not necessarily represent those of their affiliated organizations, or those of the publisher, the editors and the reviewers. Any product that may be evaluated in this article, or claim that may be made by its manufacturer, is not guaranteed or endorsed by the publisher.
