# Supplementary material for: The 2018 Revision of Italian Dietary Guidelines: Development Process, Novelties, Main Recommendations, and Policy Implications
Source: Front Nutr. 2022 Mar 25;9:861526. doi: 10.3389/fnut.2022.861526 (PMC8990302; doi:10.3389/fnut.2022.861526)
Supplement: Supplementary file 1 [file Table_1.DOCX]

Supplementary Material

# Members of the Commission of Revision of Italian Dietary Guidelines 2018

The group of experts of the Commission of Revision of Italian Dietary Guidelines 2018 was formalized by the Director-General with the official determination n. 10 of the 14 of February 2013 at the Research Center for Food and Nutrition of the Council for Agricultural Research and Economics, Rome.

**Presidents:** Andrea Ghiselli, Marcello Ticca **General Coordinator:** Laura Rossi

**Editorial Coordination Board:**

Sibilla Berni Canani, Laura Censi, Eugenio Cialfa, Amleto D’Amicis, Laura Gennaro, Andrea Ghiselli, Catherine Leclercq, Giovanni Battista Quaglia, Laura Rossi, Umberto Scognamiglio, Stefania Sette, Marcello Ticca.

**Components:**

| Rita Acquistucci | Research Center for Food and Nutrition - Council for Agricultural Research and Economics, Rome |
| --- | --- |
| Umberto Agrimi | Department of Food Safety, Nutrition and Veterinary Public Health, Italian National Institute of Health, Rome |
| Tiziana Amoriello | Research Center for Food and Nutrition - Council for Agricultural Research and Economics, Rome |
| Claudia Arganini | Research Center for Food and Nutrition - Council for Agricultural Research and Economics, Rome |
| Elena Azzini | Research Center for Food and Nutrition - Council for Agricultural Research and Economics, Rome |
| Irene Baiamonte | Research Center for Food and Nutrition - Council for Agricultural Research and Economics, Rome |
| Simona Baima | Research Center for Food and Nutrition - Council for Agricultural Research and Economics, Rome |
| Gianvincenzo Barba† | Institute of Food Sciences, National Research Council, Avellino |
| Lorenzo Barnaba | Research Center for Food and Nutrition - Council for Agricultural Research and Economics, Rome |
| Nino Carlo Battistini | Faculty of Medicine and Surgery - University of Modena and Reggio Emilia |
| Sibilla Berni Canani | Research Center for Food and Nutrition - Council for Agricultural Research and Economics, Rome |
| Furio Brighenti | Food & Drug Department, Human Nutrition Unit, University of Parma |
| Pasquale Buonocore | Research Center for Food and Nutrition - Council for Agricultural Research and Economics, Rome |
| Giulia Cairella | Italian Society of Human Nutrition, Prevention Department, Local Health Authority Rome 2, Rome |
| Emanuela Camilli | Research Center for Food and Nutrition - Council for Agricultural Research and Economics, Rome |
| Raffaella Canali | Research Center for Food and Nutrition - Council for Agricultural Research and Economics, Rome |
| Lucio Capurso | Director Emeritus Gastrointestinal Unit, S Filippo Neri Hospital, Rome |
| Marina Carbonaro | Research Center for Food and Nutrition - Council for Agricultural Research and Economics, Rome |
| Marina Carcea | Research Center for Food and Nutrition - Council for Agricultural Research and Economics, Rome |
| Michele Carruba | Center for Study and Research on Obesity, Department of Biomedical Technology and Translational Medicine, Università degli Studi di Milano (University of Milan) |
| Giovina Catasta | Research Center for Food and Nutrition - Council for Agricultural Research and Economics, Rome |
| Laura Censi | Research Center for Food and Nutrition - Council for Agricultural Research and Economics, Rome |
| Eugenio Cialfa | formerly National Research Institute on Food and Nutrition, Rome |
| Donatella Ciarapica | Research Center for Food and Nutrition - Council for Agricultural Research and Economics, Rome |
| Francisco Javier Comendador | Research Center for Food and Nutrition - Council for Agricultural Research and Economics, Rome |
| Claudio Cricelli | Chairman, The Italian Society of General practitioners and Primary Care Professionals, Florence |
| Massimo Cuzzolaro | formerly University of Roma Sapienza. Editor in Chief di Eating and Weight Disorders Studies on Anorexia, Bulimia and Obesity |
| Amleto D'Amicis | formerly National Research Institute on Food and Nutrition, Rome |
| Valeria del Balzo | Food Science and Human Nutrition, Department of Experimental Medicine, University of Roma Sapienza |
| Eugenio Del Toma | Italian Association of Dietetics and Clinical Nutrition, Rome |
| Chiara Devirgiliis | Research Center for Food and Nutrition - Council for Agricultural Research and Economics, Rome |
| Gabriella Di Lena | Research Center for Food and Nutrition - Council for Agricultural Research and Economics, Rome |
| Massimiliano Dona | National Consumer Union, Rome |
| Lorenzo Maria Donini | Food Science and Human Nutrition, Department of Experimental Medicine University of Roma Sapienza |
| Alessandra Durazzo | Research Center for Food and Nutrition - Council for Agricultural Research and Economics, Rome |
| Marika Ferrari | Research Center for Food and Nutrition - Council for Agricultural Research and Economics, Rome |
| Gaetana Ferri | Ministry of Health, Directorate-General for hygiene, food safety and nutrition, Rome |
| Alberto Finamore | Research Center for Food and Nutrition - Council for Agricultural Research and Economics, Rome |
| Giuseppe Fatati | Unit of Diabetology, Dietetics and Clinical Nutrition, S. Maria Hospital, Terni |
| Myriam Galfo | Research Center for Food and Nutrition - Council for Agricultural Research and Economics, Rome |
| Claudio Galli† | Department of Pharmacological and Biomolecular Sciences, Università degli Studi di Milano (University of Milan) |
| Laura Gennaro | Research Center for Food and Nutrition - Council for Agricultural Research and Economics, Rome |
| Andrea Ghiselli | Research Center for Food and Nutrition - Council for Agricultural Research and Economics, Rome |
| Rosalba Giacco | Institute of Food Sciences, National Research Council, Avellino, Italy |
| Michelangelo Giampietro | Professor at the Sports School of the Italian National Olympic Committee – Rome, Italy. Sports Medicine Service, Local Health Authority Viterbo and Rome 2 |
| Marcello Giovannini | Italian Society of Pediatric Nutrition, Department of Pediatrics, Università degli Studi di Milano (University of Milan) |
| Emilia Guberti | MD Chief-Medical-Officer, Food Safety and Nutrition Service (FSNS), Public Health Authority – Bologna, Coordinator of “FSNS” national teamwork of Società Italiana di Igiene Medicina Preventiva e Sanità Pubblica (SItI) - Rome. |
| Federica Intorre | Research Center for Food and Nutrition - Council for Agricultural Research and Economics, Rome |
| Carlo La Vecchia | Department of Clinical Sciences and Community Health, Università degli Studi di Milano (University of Milan) |
| Catherine Leclercq | Food and Agriculture Organization of the United Nations (FAO), in leave of absence from the Research Center for Food and Nutrition - Council for Agricultural Research and Economics, Rome |
| Cinzia Le Donne | Research Center for Food and Nutrition - Council for Agricultural Research and Economics, Rome |
| Rosa Lenoci | President of Association of Italian Nutrition Biologists |
| Francesco Leonardi | Food Education Italy Foundation, Milan |
| Ginevra Lombardi Boccia | Research Center for Food and Nutrition - Council for Agricultural Research and Economics, Rome |
| Caterina Lombardo | Department of Psychology, University of Roma Sapienza |
| Massimo Lucarini | Research Center for Food and Nutrition - Council for Agricultural Research and Economics, Rome |
| Sabrina Lucchetti | Research Center for Food and Nutrition - Council for Agricultural Research and Economics, Rome |
| Lucio Lucchin | Italian Association of Dietetics and Clinical Nutrition, Medical Director of the Clinical Nutrition Unit Sanitary District of Bolzano |
| Pamela Manzi | Research Center for Food and Nutrition - Council for Agricultural Research and Economics, Rome |
| Stefania Marconi | Research Center for Food and Nutrition - Council for Agricultural Research and Economics, Rome |
| Luisa Marletta | Research Center for Food and Nutrition - Council for Agricultural Research and Economics, Rome |
| Deborah Martone | Research Center for Food and Nutrition - Council for Agricultural Research and Economics, Rome |
| Maria Mattera | Research Center for Food and Nutrition - Council for Agricultural Research and Economics, Rome |
| Francesca Melini | Research Center for Food and Nutrition - Council for Agricultural Research and Economics, Rome |
| Elena Mengheri | Research Center for Food and Nutrition - Council for Agricultural Research and Economics, Rome |
| Pietro Antonio Migliaccio | Italian Society of Food Science and Nutrition, Rome |
| Elisabetta Moneta | Research Center for Food and Nutrition - Council for Agricultural Research and Economics, Rome |
| Lorenzo Morelli | Dean of the Department of Agricultural, Food and Environmental Sciences Università Cattolica del Sacro Cuore Piacenza - Cremona |
| Valentina Narducci | Research Center for Food and Nutrition - Council for Agricultural Research and Economics, Rome |
| Fausta Natella | Research Center for Food and Nutrition - Council for Agricultural Research and Economics, Rome |
| Elena Orban | Research Center for Food and Nutrition - Council for Agricultural Research and Economics, Rome |
| Sergio Pacini† | Past President Association of Italian Nutrition Biologists |
| Flavio Paoletti | Research Center for Food and Nutrition - Council for Agricultural Research and Economics, Rome |
| Lucia Paris | Directorate general for sustainable growth and quality of development, Ministry of Ecological Transition, Rome |
| Andrea Poli | Italian Society for the Study of Arteriosclerosis, Milan |
| Pierluigi Pecoraro | Nutrition Unit, Department of Prevention, Local Health Authority Napoli 3 Sud, Naples |
| Marina Peparaio | Research Center for Food and Nutrition - Council for Agricultural Research and Economics, Rome |
| Giuditta Perozzi | Research Center for Food and Nutrition - Council for Agricultural Research and Economics, Rome |
| Raffaela Piccinelli | Research Center for Food and Nutrition - Council for Agricultural Research and Economics, Rome |
| Angela Polito | Research Center for Food and Nutrition - Council for Agricultural Research and Economics, Rome |
| Marisa Porrini | Department of Food, Environmental and Nutritional Sciences, Università degli Studi di Milano (University of Milan). |
| Giovanni Battista Quaglia | formerly National Research Institute on Food and Nutrition, Rome |
| Antonia Ricci | Istituto Zooprofilattico Sperimentale delle Venezie, Padua |
| Antonio Raffo | Research Center for Food and Nutrition - Council for Agricultural Research and Economics, Rome |
| Giulia Ranaldi | Research Center for Food and Nutrition - Council for Agricultural Research and Economics, Rome |
| Romana Roccaldo | Research Center for Food and Nutrition - Council for Agricultural Research and Economics, Rome |
| Marianna Roselli | Research Center for Food and Nutrition - Council for Agricultural Research and Economics, Rome |
| Laura Rossi | Research Center for Food and Nutrition - Council for Agricultural Research and Economics, Rome |
| Stefania Ruggeri | Research Center for Food and Nutrition - Council for Agricultural Research and Economics, Rome |
| Anna Saba | Research Center for Food and Nutrition - Council for Agricultural Research and Economics, Rome |
| Yula Sambuy | Research Center for Food and Nutrition - Council for Agricultural Research and Economics, Rome |
| Cristina Scaccini | Research Center for Food and Nutrition - Council for Agricultural Research and Economics, Rome |
| Luca Scalfi | Department of Public Health, School of Medicine, Federico II University, Naples |
| Emanuele Scafato | Director, National Observatory on Alcohol, WHO Collaborating Centre for Research & Health Promotion on Alcohol and Alcohol-Related Health Problems, National Centre on Addictions and Doping, Italian National Institute of Health, Rome |
| Maria Laura Scarino | Research Center for Food and Nutrition - Council for Agricultural Research and Economics, Rome |
| Umberto Scognamiglio | Research Center for Food and Nutrition - Council for Agricultural Research and Economics, Rome |
| Mauro Serafini | Functional Food and Metabolic Stress Prevention Laboratory, Teramo University |
| Stefania Sette | Research Center for Food and Nutrition - Council for Agricultural Research and Economics, Rome |
| Marco Silano | Italian National Institute of Health, Unit of Human Nutrition and Health, Rome |
| Fiorella Sinesio | Research Center for Food and Nutrition - Council for Agricultural Research and Economics, Rome |
| Angela Spinelli | National Center for Disease Prevention and Health Promotion; Italian National Institute of Health, Rome |
| Pasquale Strazzullo | Italian Society of Human Nutrition, Federico II University of Naples |
| Marcello Ticca | formerly National Research Institute on Food and Nutrition, Rome |
| Elisabetta Toti | Research Center for Food and Nutrition - Council for Agricultural Research and Economics, Rome |
| Ersilia Troiano | Italian Association of Dietitians, Rome |
| Valeria Turfani | Research Center for Food and Nutrition - Council for Agricultural Research and Economics, Rome |
| Aida Turrini | Research Center for Food and Nutrition - Council for Agricultural Research and Economics, Rome |
| Eugenia Venneria | Research Center for Food and Nutrition - Council for Agricultural Research and Economics, Rome |
| Alessandro Vienna | Ministry of Education, Universities, and Research, Rome |
