# Supplementary figures and images for: The 2018 Revision of Italian Dietary Guidelines: Development Process, Novelties, Main Recommendations, and Policy Implications
Source: Front Nutr. 2022 Mar 25;9:861526. doi: 10.3389/fnut.2022.861526 (PMC8990302; doi:10.3389/fnut.2022.861526)

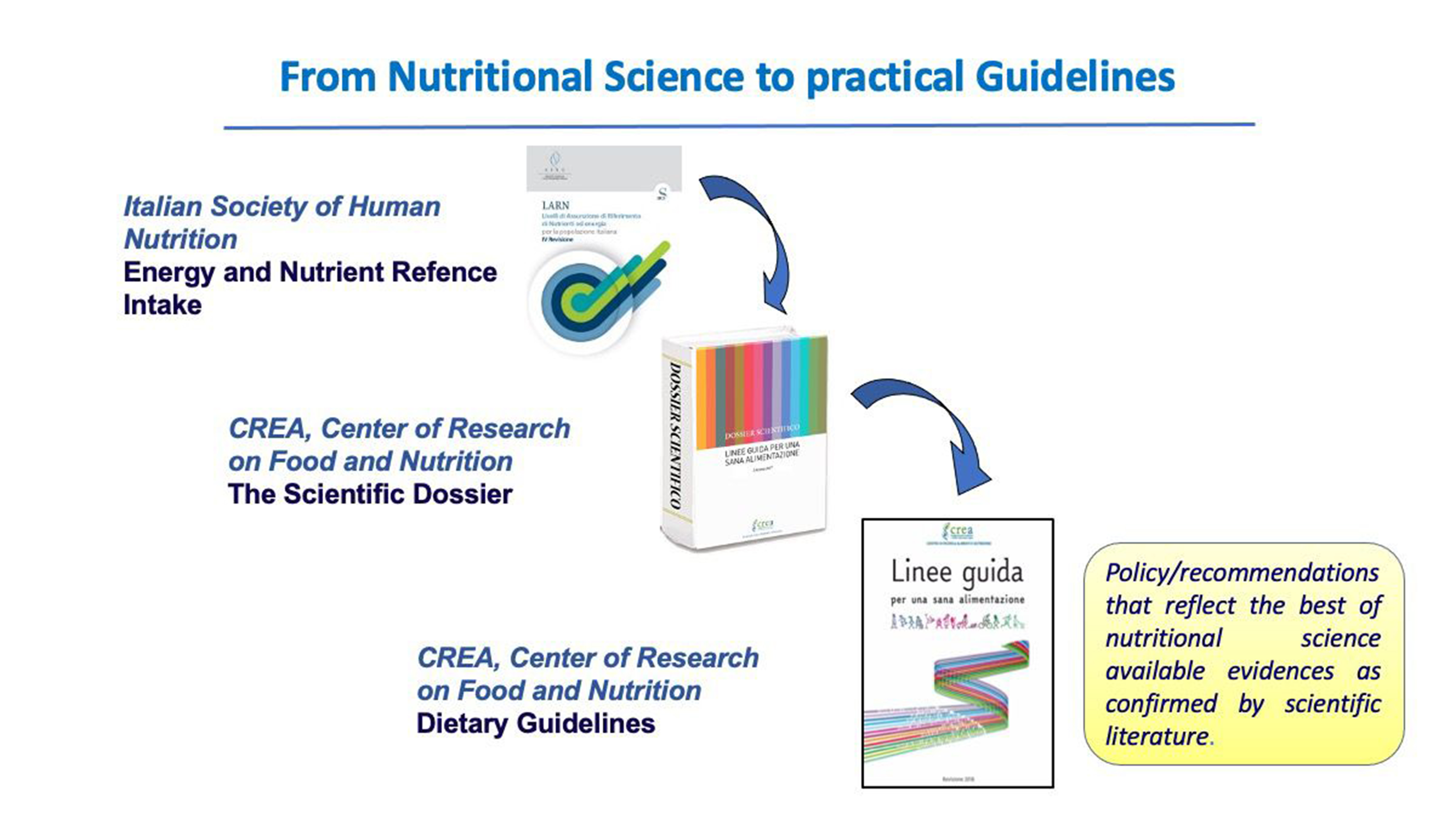

Supplement: Supplementary file 2 [file Image_1.JPEG]
